# Development and Validation of a Deep Learning Model to Predict the Occurrence and Severity of Retinopathy of Prematurity

**DOI:** 10.1001/jamanetworkopen.2022.17447

**Published:** 2022-06-16

**Authors:** Qiaowei Wu, Yijun Hu, Zhenyao Mo, Rong Wu, Xiayin Zhang, Yahan Yang, Baoyi Liu, Yu Xiao, Xiaomin Zeng, Zhanjie Lin, Ying Fang, Yijin Wang, Xiaohe Lu, Yanping Song, Wing W. Y. Ng, Songfu Feng, Honghua Yu

**Affiliations:** 1 Guangdong Eye Institute, Department of Ophthalmology, Guangdong Provincial People’s Hospital, Guangdong Academy of Medical Sciences, Guangzhou, China; 2 Department of Ophthalmology, General Hospital of Central Theater Command, Wuhan, China; 3 Guangdong Provincial Key Laboratory of Computational Intelligence and Cyberspace Information, School of Computer Science and Engineering, South China University of Technology, Guangzhou, China; 4 Department of Ophthalmology, Zhujiang Hospital of Southern Medical University, Guangzhou, China; 5 State Key Laboratory of Ophthalmology, Zhongshan Ophthalmic Center, Sun Yat-sen University, Guangzhou, China; 6 Department of Neonatology, Second Nanning People’s Hospital, Nanning, China

## Abstract

**Importance:**

Retinopathy of prematurity (ROP) is the leading cause of childhood blindness worldwide. Prediction of ROP before onset holds great promise for reducing the risk of blindness.

**Objective:**

To develop and validate a deep learning (DL) system to predict the occurrence and severity of ROP before 45 weeks’ postmenstrual age.

**Design, Setting, and Participants:**

This retrospective prognostic study included 7033 retinal photographs of 725 infants in the training set and 763 retinal photographs of 90 infants in the external validation set, along with 46 characteristics for each infant. All images of both eyes from the same infant taken at the first screening were labeled according to the final diagnosis made between the first screening and 45 weeks’ postmenstrual age. The DL system was developed using retinal photographs from the first ROP screening and clinical characteristics before or at the first screening in infants born between June 3, 2017, and August 28, 2019.

**Exposures:**

Two models were specifically designed for predictions of the occurrence (occurrence network [OC-Net]) and severity (severity network [SE-Net]) of ROP. Five-fold cross-validation was applied for internal validation.

**Main Outcomes and Measures:**

Area under the receiver operating characteristic curve (AUC), accuracy, sensitivity, and specificity to evaluate the performance in ROP prediction.

**Results:**

This study included 815 infants (450 [55.2%] boys) with mean birth weight of 1.91 kg (95% CI, 1.87-1.95 kg) and mean gestational age of 33.1 weeks (95% CI, 32.9-33.3 weeks). In internal validation, mean AUC, accuracy, sensitivity, and specificity were 0.90 (95% CI, 0.88-0.92), 52.8% (95% CI, 49.2%-56.4%), 100% (95% CI, 97.4%-100%), and 37.8% (95% CI, 33.7%-42.1%), respectively, for OC-Net to predict ROP occurrence and 0.87 (95% CI, 0.82-0.91), 68.0% (95% CI, 61.2%-74.8%), 100% (95% CI, 93.2%-100%), and 46.6% (95% CI, 37.3%-56.0%), respectively, for SE-Net to predict severe ROP. In external validation, the AUC, accuracy, sensitivity, and specificity were 0.94, 33.3%, 100%, and 7.5%, respectively, for OC-Net, and 0.88, 56.0%, 100%, and 35.3%, respectively, for SE-Net.

**Conclusions and Relevance:**

In this study, the DL system achieved promising accuracy in ROP prediction. This DL system is potentially useful in identifying infants with high risk of developing ROP.

## Key Points

**Question:**

Can a deep learning system provide reliable prediction of retinopathy of prematurity (ROP) using retinal photographs and clinical characteristics?

**Findings:**

In this prognostic study including data from 815 infants, the mean areas under the receiver operating characteristic curve (AUCs) of the system were 0.90 and 0.87 in predicting the occurrence and severity of ROP, respectively. For the external validation set, the AUCs were 0.94 and 0.88, respectively.

**Meaning:**

These findings suggest the feasibility of using deep learning approaches to predict ROP with high accuracy and generalizability.

## Teaser

This prognostic study validates a deep learning system to predict the occurrence and severity of retinopathy of prematurity (ROP) before 45 weeks’ postmenstrual age.

## Introduction

The reduction of blindness in children is one of the major goals of the World Health Organization’s Vision 2020 program.^1^ As the leading cause of childhood blindness, retinopathy of prematurity (ROP) is estimated to affect more than 184 700 children worldwide.^2^ The incidence and severity of ROP are associated with various factors, such as birth weight (BW), gestational age (GA), oxygen exposure, blood transfusions, and several systemic risk factors.^3,4,5,6,7,8,9,10^ Early screening can effectively reduce childhood blindness and health care costs associated with ROP.^11,12,13,14,15^ Because of the lack of widespread implementation of a cost-effective screening strategy, the cost of ROP screening to the global health system remains high.^12,14^ While the window for treating ROP is brief, treatment has multigenerationally beneficial consequences for individuals, families, societies, and national economies.^11,13,14^ Thus, early screening, regular monitoring, and timely treatment are critical for premature infants.

Multiple longitudinal examinations using fundus photography (eg, by RetCam3) have been widely adopted for ROP screening, which are stressful to infants, laborious to ophthalmologists, and often unavailable in remote areas.^16,17^ There is also a growing shortage of pediatric ophthalmologists or retinal specialists capable of performing ROP screening.^18^ Moreover, less than 10% of mild ROP cases progress to sight-threatening ROP that requires further treatment.^16,19^ Given the extensive efforts required for ROP screening, low rates of treatment-requiring ROP, and misdiagnosis of severe ROP because of interrupted follow-up, cost-effective programs that can identify infants with high risk of severe ROP are needed.^16,20,21,22,23^ Previous studies have used traditional regression analysis to predict ROP based on BW, GA, gender, and/or postnatal weight gain, among other factors.^16,20,21,22,23,24^ However, some of these prediction models are flawed because of they neglect retinal status, which is also important for ROP prediction.^25,26,27^

Deep learning (DL) has been recently used for the automated diagnosis of many ocular diseases.^28,29,30,31^ Several DL systems have been developed for automated diagnosis of the plus disease of ROP with high accuracy (>89.6%) based on RetCam images.^19,32,33,34^ However, to our knowledge, there is no DL system to date capable of predicting the occurrence and severity of ROP. In the present study, we developed a DL system for the prediction of occurrence and severity of ROP before 45 weeks’ postmenstrual age (PMA) based on retinal photographs from the first ROP screening and clinical characteristics before or at the first ROP screening.

## Methods

### Data Set Preparation

**Figure 1.**
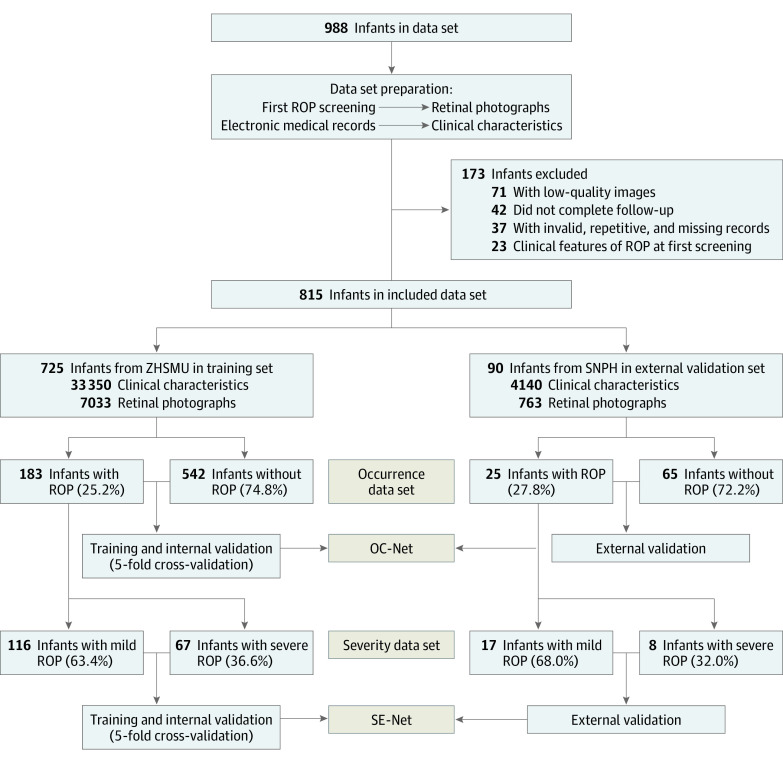
Workflow Diagram ROP indicates retinopathy of prematurity; SNPH, Second Nanning People’s Hospital; and ZHSMU, Zhujiang Hospital of Southern Medical University.

The study flowchart is presented in Figure 1. We collected data retrospectively on 988 infants (delivered between June 3, 2017, and August 28, 2019) who underwent ROP examinations at the Zhujiang Hospital of Southern Medical University (ZHSMU) and the Second Nanning People’s Hospital (SNPH). The ROP screening criteria were developed according to an international guideline,^35^ including a BW of less than 1501 g, a GA of 30 weeks or younger, or risk for ROP as determined by the pediatrician or neonatologist.^35^ The study was conducted according to the Declaration of Helsinki^36^ and was approved by the Research Ethics Committee of ZHSMU and SNPH. Informed consent was obtained from parents of all infants enrolled. This study followed the Transparent Reporting of a Multivariable Prediction Model for Individual Prognosis or Diagnosis (TRIPOD) reporting guideline.

In total, data of 988 infants who underwent ROP screening were retrieved from the screening centers and electronic medical records (EMR) in ZHSMU and SNPH. Of those, 71 infants (7.2%) were excluded because retinal photographs were labeled as unacceptable for evaluation; 37 infants (3.7%) were excluded for invalid, repetitive, and missing records; 42 infants (4.3%) were excluded for failing to complete follow-up; and 23 infants (2.3%) were excluded for having clinical features of ROP at the first screening. There were 815 infants (82.5%) with complete data and eligible for the development and validation of the DL system who had a known ROP outcome before 45 weeks’ PMA but no clinical features of ROP on the retinal photographs at the first screening. There was no significant difference in the birth characteristics between the included and excluded infants (eTable 1 in the Supplement). A total of 7033 images from the first screening of 725 infants were collected from ZHSMU by certified ophthalmologists and used for training and cross-validating the DL system. An additional 763 images from the first screening of 90 infants were obtained from SNPH following the same criteria of the training set and were used for external validation. A total of 46 clinical characteristics for each infant were also collected from EMR in ZHSMU and SNPH before or at the first screening. Screening schedule details performed at each follow-up are reported in eAppendix 1 in the Supplement.

During each screening, multiple retinal photographs are captured using a commercially available camera (RetCam; Natus Medical Inc). A total of 46 clinical characteristics of each infant from the EMR were extracted, including 7 maternal factors, 18 neonatal factors, 7 treatment factors, and 14 laboratory factors. These clinical characteristics were selected according to the reported risk factors associated with the development of ROP.^3,5,7,9,10,21,37,38,39,40,41,42,43,44,45,46,47,48,49,50,51^ Detailed definitions, the time of collection, and references to related studies of the characteristics are provided in eAppendix 2 in the Supplement.

### Image Labeling

All retinal photographs used for the development and validation of the DL system were collected only from the first screening. All cases were annotated independently by 2 ophthalmologists (S.F. and Q.W.) with more than 3 years’ experience of ROP care according to the retinal photographs. Since the ROP prediction was performed on each case rather than each retinal photograph independently, all retinal photographs of both eyes from the same infant were defined and labeled as 1 case. A case would be excluded if 1 of 2 ophthalmologists labeled the quality of the retinal photographs unacceptable for evaluation. All ophthalmoscopic and image-based examination findings for each case were documented according to the international classification of ROP, with zone, stage, and plus disease.^52^ A case was annotated ROP if the clinical features of ROP were found in any retinal photographs of this case; otherwise, it was annotated normal. To predict treatment-requiring ROP, cases with type II ROP; zone II stage 1 or stage 2 ROP without plus disease; and zone III stage 1, 2, or 3 ROP were graded as mild ROP, which only requires regular follow-up; cases with threshold disease, stage 4 or 5 ROP, and type I or aggressive posterior–ROP were graded as severe ROP, which needs prompt treatment.^35,53^ If a case had different severities in each eye, the case would be graded according to the higher ROP grade of either eye.

The κ was 0.81 for annotation of ROP occurrence and 0.72 for annotation of severe ROP, suggesting good agreement of the 2 clinical ophthalmologists in image labeling.^54^ Moreover, the labels were double confirmed by a senior retinal specialist (H.Y.) with more than 15 years of clinical retina experience to generate a final annotation. These annotations were used as the ground-truth labels in the training and validation of the DL system.

### DL System Development and Validation

**Figure 2.**
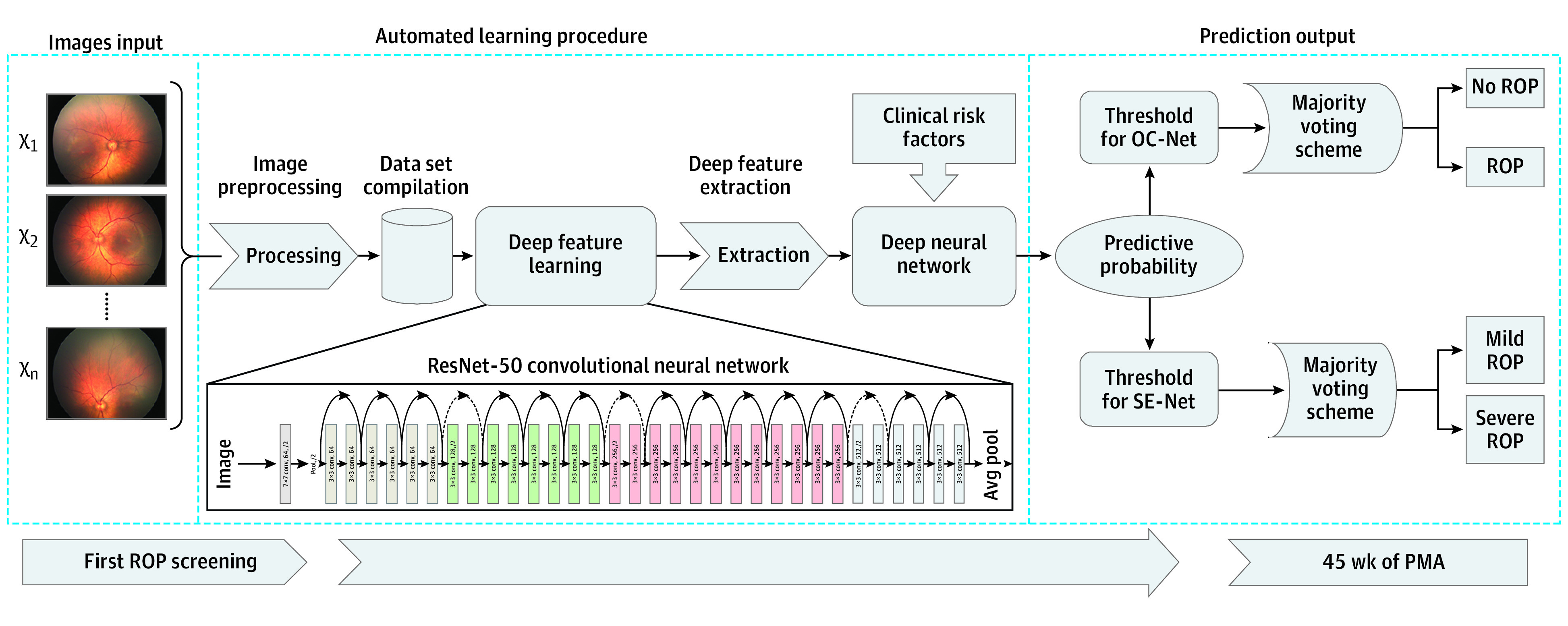
Illustration of the Proposed Algorithmic Pipeline A deep learning system was developed to predict occurrence and severity of retinopathy of prematurity (ROP) before 45 weeks’ postmenstrual age (PMA) using a major voting scheme. OC-Net indicates occurrence network; ResNet, residual network; and SE-Net, severity network.

An illustration of our DL system is presented in Figure 2. The ROP prediction consists of 2 tasks. One is to predict ROP occurrence before 45 weeks’ PMA; the other is to predict severity of ROP. An occurrence network (OC-Net) and a severity network (SE-Net) with similar network architectures were developed for these 2 tasks. The prediction labels used in OC-Net were *normal* and *ROP*, and the prediction labels used in SE-Net were *mild ROP* and *severe ROP*.

Multiple preprocessing steps were performed to standardize input images before deep feature learning. After preprocessing and characterization by the residual network–50 (Microsoft Corp), 512 features of each retinal photograph, extracted from the global average pool layer, were concatenated with 46 clinical characteristics of the same case into a final vector of 558 dimensions. A deep neural network was trained on the representative feature vector of 558 values to generate the predictive probability of each retinal photograph.

We adopted 3 different training schemes (ie, majority voting, 1-vote veto, and image-level methods) to output the predictive labels based on the probability threshold. Details about the development and validation of the DL system and the different training schemes are provided in eAppendix 3 in the Supplement.

For internal validation of the 3 training schemes, the 5-fold cross-validation manner was used on the training set, including the occurrence data set and the severity data set. The mean and 95% CI of the performing metrics of the 5 runs were recorded to measure the overall performance of the 3 training schemes. Then, the best of the 3 training schemes was deployed in the DL system for further validation, with a requirement of 100% sensitivity. OC-Net and SE-Net with 100% sensitivity were used to perform the external validation. Furthermore, we obtained all weights of each characteristic from the last fully connected layers and calculated weight ratios of all characteristics by normalization to reflect the importance of characteristics to the DL system. The higher the weight ratios are, the more important the corresponding characteristics are for the prediction task.

### ROP Prediction Using ROPScore

To better demonstrate the prediction accuracy of our DL system, performance of a commonly used algorithm, ROPScore, was also evaluated. ROPScore is accessible online and also used to predict occurrence and severity of ROP for each infant before 45 weeks’ PMA.^23,24^ Details about the validation using ROPScore are provided in eAppendix 4 in the Supplement. The predictive performance of ROPScore was compared with the DL system.

### Visualization Method

To visualize the critical regions in retinal photographs highly associated with the ROP prediction, Grad-CAM was used to generate saliency maps and enhance the interpretability of the DL system.^55^ Grad-CAM calculates feature weight to generate heat maps that highlight the regions that play an important role in the final prediction.^55^ By visualizing the saliency maps, we can observe and determine the contribution of potential fundus features to the final ROP prediction.

### Statistical Analysis

Breakdowns of the predictive labels with reference to the ground-truth labels were depicted as confusion matrices, which were used to calculate the accuracy, sensitivity, and specificity of OC-Net and SE-Net with the 3 training schemes (Python version 3.7.0 [Python Software Foundation]). We also used the area under the receiver operating characteristic curve (AUC) to determine the predictive performance of the DL system with reference to the ground-truth labels and conducted calibration plots to examine overestimation or underestimation of risks in the DL system.^21^

## Results

### Description of the Study Cohort

Among 815 included infants, 450 (55.2%) were boys, the mean GA was 33.1 weeks (95% CI, 32.9-33.3 weeks; median, 33 weeks; range, 25-40 weeks), and the mean BW was 1.91 kg (95% CI, 1.87-1.95 kg; median, 1.92 kg; range, 0.75-3.80 kg) (eFigure 1 in the Supplement). Demographic characteristics of the training and the external validation sets are summarized in Table 1.

**Table 1.** Demographic Characteristics

Characteristics	No. (%)		
	All (N = 815)	Training set (n = 725)	External validation set (n = 90)
Maternal factors			
Maternal age, mean (SD), y	30.9 (5.6)	30.9 (5.6)	31.2 (5.7)
Cesarean delivery	445 (54.6)	392 (54.1)	53 (58.9)
IVF-ET	98 (12.0)	79 (10.9)	19 (21.1)
Gestational hypertension	76 (9.3)	63 (8.7)	13 (14.4)
Gestational diabetes	154 (18.9)	131 (18.1)	23 (25.6)
Intrauterine infection	26 (3.2)	24 (3.3)	2 (2.2)
Use of dexamethasone	160 (19.6)	142 (19.6)	18 (20.0)
Neonatal factors			
Gestational age, mean (SD), wk	33.1 (3.2)	33.4 (3.2)	30.7 (2.2)
Birth weight, mean (SD), kg	1.91 (0.6)	2.0 (0.6)	1.5 (0.3)
Male sex	450 (55.2)	401 (55.3)	50 (55.6)
Small for gestational age	61 (7.5)	54 (7.5)	10 (11.1)
Multiple gestations	202 (24.8)	175 (24.1)	27 (30.0)
Apgar Score, mean (SD)			
1-min	8.5 (1.9)	8.5 (1.9)	8.6 (1.5)
5-min	9.4 (1.3)	9.3 (1.3)	9.4 (0.9)
Intrauterine distress	81 (9.9)	75 (10.3)	6 (6.7)
Asphyxia	140 (17.2)	126 (17.4)	14 (15.6)
Bronchopulmonary dysplasia	148 (18.2)	121 (16.7)	27 (30.0)
Intracranial hemorrhage	166 (20.4)	139 (19.2)	27 (30.0)
Sepsis	103 (12.6)	89 (12.8)	14 (15.6)
Hypoxic ischemic encephalopathy	77 (9.5)	76 (10.5)	8 (8.9)
Respiratory distress syndrome	334 (41.0)	266 (36.7)	36 (40.0)
Pneumonia	430 (52.8)	359 (49.5)	47 (52.2)
Necrotizing enterocolitis	47 (5.8)	41 (5.7)	6 (6.7)
Neonatal jaundice	578 (70.9)	504 (69.5)	73 (81.1)
Patent ductus arteriosus	229 (28.1)	197 (27.2)	33 (36.7)
Treatment factors			
History of oxygen exposure	560 (68.7)	497 (68.6)	63 (70.0)
Use of oxygen in MV	422 (51.8)	366 (50.5)	56 (62.2)
Duration of oxygen in MV, mean (SD), d	15.5 (20.0)	14.3 (19.4)	25.3 (22.1)
Blood transfusion	482 (59.1)	423 (58.3)	59 (65.6)
RBC transfusion	423 (51.9)	367 (50.6)	56 (62.2)
No. of RBC transfusions, mean (SD)	1.1 (1.6)	1.1 (1.6)	1.6 (1.9)
Volume of RBC transfusion, mean (SD), U	0.4 (0.6)	0.4 (0.6)	0.5 (0.6)
Laboratory factors			
High-sensitivity CRP, mean (SD), mg/dL	0.7 (2.3)	0.8 (2.4)	0.3 (1.0)
WBC count, mean (SD), /μL	11 900 (6800)	12 200 (7000)	9900 (4900)
RBC count, mean (SD), 10^6^/μL	4.3 (1.1)	4.3 (1.0)	4.6 (1.4)
Hemoglobin concentration, mean (SD), g/dL	14.97 (3.68)	14.82 (3.54)	16.17 (4.53)
Hematocrit, mean (SD), mg/L	0.4 (0.1)	0.4 (0.1)	0.5 (1.0)
Corpuscular volume, mean (SD), fL	101.4 (11.5)	100.9 (11.6)	104.9 (10.5)
RBC distribution width, mean (SD), %	16.7 (2.3)	16.7 (2.4)	16.7 (1.5)
Absolute neutrophil count, mean (SD), /μL	7000 (5600)	7200 (5700)	5400 (3700)
Absolute lymphocyte count, mean (SD), /μL	3600 (3100)	3600 (3200)	3200 (1500)
Absolute monocyte count, mean (SD), /μL	1300 (1200)	1400 (1300)	1200 (1200)
Platelet count, mean (SD), 10^3^/μL	261.6 (122.9)	262.9 (127.2)	251.2 (80.0)
Platelet volume, mean (SD), fL	10.3 (1.1)	10.3 (1.1)	10.2 (0.8)
Platelet distribution width, mean (SD), %	12.3 (2.5)	12.4 (2.6)	11.4 (1.7)
Serum total bilirubin, mean (SD), mg/dL	5.99 (4.13)	5.97 (4.27)	6.10 (2.70)

Abbreviations: CRP, C-reactive protein; IVF-ET, in vitro fertilization and embryo transfer; MV, mechanical ventilation; RBC, red blood cell; WBC, white blood cell.

SI conversion factors: To convert CRP to milligrams per liter, multiply by 10; hemoglobin to grams per liter, multiply by 10.0; lymphocyte count, monocyte count, neutrophil count, and WBC to ×10^9^ cells per liter, multiply by 0.001; platelet count to ×10^9^ cells per liter, multiply by 1; RBC to ×10^12^ cells per liter, multiply by 1; and total bilirubin to micromoles per liter, multiply by 17.104.

### Internal Validation Under Requirement of 100% Sensitivity

Of the 725 cases included in the training set, 542 (74.8%) were annotated normal, and 183 (25.2%) were annotated ROP. Of the 183 ROP cases, 116 (63.4%) were graded mild ROP, and 67 (36.6%) were graded severe ROP. The results of the 3 training schemes of OC-Net and SE-Net are summarized in eTable 2 in the Supplement. Among the 3 training schemes, the major voting scheme achieved the best overall performance for both OC-Net and SE-Net. Thus, the major voting scheme was deployed for further validation under the requirement of 100% sensitivity. After adjusting thresholds of OC-Net to 0.10 and SE-Net to 0.26, both OC-Net and SE-Net achieved 100% sensitivity to capture all ROP and severe ROP cases, respectively, with the same model structure.^21,22^

**Figure 3.**
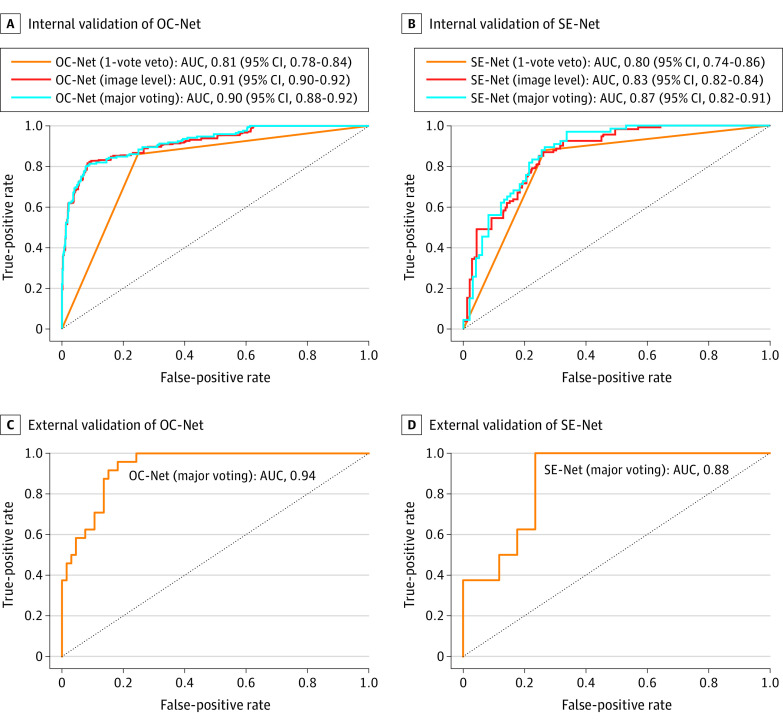
Receiver Operating Characteristic Curve for Evaluating the Predictive Ability of the Deep Learning System AUC indicates area under the receiver operating characteristic curve; OC-Net, occurrence network; and SE-net, severity network.

Applying the threshold of 0.10, the mean AUC, accuracy, sensitivity, and specificity of OC-Net to predict ROP occurrence were 0.90 (95% CI, 0.88-0.92), 52.8% (95% CI, 49.2%-56.4%), 100% (95% CI, 97.4%-100%), and 37.8% (95% CI, 33.7%-42.1%), respectively (Table 2 and Figure 3A). Applying the threshold of 0.26, the mean AUC, accuracy, sensitivity, and specificity of SE-Net to predict severe ROP were 0.87 (95% CI, 0.82-0.91), 68.0% (95% CI, 61.2%-74.8%), 100% (95% CI, 93.2%-100%), and 46.6% (95% CI, 37.3%-56.0%), respectively (Table 2 and Figure 3B). Moreover, calibration plots of the major voting scheme showed that the model was well adapted (eFigure 3 in the Supplement), and improvement in the DL system performance by clinical characteristics is described in eAppendix 5 in the Supplement.

**Table 2.** Overall Performance of the Deep Learning System Under the Requirement of 100% Sensitivity and ROPScore in Prediction of ROP

System	AUC (95% CI)	% (95% CI)		
		Accuracy	Sensitivity	Specificity
**Internal validation for prediction of ROP occurrence, with threshold of 0.10**				
OC-Net, major voting scheme	0.90 (0.88-0.92)	52.8 (49.2-56.4)	100 (97.4-100)	37.8 (33.7-42.1)
ROPScore	0.76 (0.72-0.80)	69.0 (65.6-72.3)	61.7 (54.3-68.7)	71.4 (67.4-75.1)
**Internal validation for prediction of ROP severity, with threshold of 0.26**				
SE-Net, major voting scheme	0.87 (0.82-0.91)	68.0 (61.2-74.8)	100 (93.2-100)	46.6 (37.3-56.0)
ROPScore	0.67 (0.59-0.75)	67.8 (64.4-71.2)	53.7 (41.2-65.8)	75.9 (66.9-83.1)
**External validation for prediction of ROP occurrence, with threshold of 0.10 **				
OC-Net, major voting scheme	0.94	33.3	100	7.5
ROPScore	0.76 (0.72-0.80)	58.9 (55.3-62.5)	100 (83.4-100)	43.1 (31.1-55.9)
**External validation for prediction of ROP severity, with threshold of 0.26**				
SE-Net, major voting scheme	0.88	56.0	100	35.3
ROPScore	0.80 (0.62-0.98)	60.0 (56.4-63.6)	100 (59.8-100)	41.2 (19.4-66.5)

Abbreviations: AUC, area under the receiver operating characteristic curve; OC-Net, occurrence-network; ROP, retinopathy of prematurity; SE-Net, severity-network.

### External Validation

Of the 90 cases included in the external validation set, 65 (72.2%) were annotated normal, and 25 (27.8%) were annotated ROP. Of the 25 ROP cases, 17 (68.0%) were graded mild ROP, and 8 (32.0%) were graded severe ROP. Using the same threshold as internal validation, the AUC, accuracy, sensitivity, and specificity were 0.94, 33.3%, 100%, and 7.5%, respectively, for OC-Net and 0.88, 56.0%, 100%, and 35.3%, respectively, for SE-Net (Table 2 and Figure 3).

### Characteristics of Infants With False-Negative and True-Positive Results for the Prediction of ROP

To explore the characteristics of cases with incorrect predictions, we compared the birth characteristics between the false-negative and true-positive results of occurrence and severity predictions for ROP. For OC-Net, the major voting scheme predicted that 21 infants (10.1%) with ROP would not have ROP, while 187 infants (89.9%) infants with ROP were correctly predicted. Compared with infants with true-positive results, those with false-negative results were more likely to be born via cesarean delivery with a later GA, greater BW, better systemic condition, and less need for blood transfusions and oxygen therapy (eTable 3 in the Supplement). For SE-Net, the major voting scheme predicted that 10 infants (13.3%) with severe ROP would have mild ROP, while 65 infants (86.7%) with severe ROP were correctly predicted. Compared with those with true-positive results, the infants with false-positive results had greater BW, lower prevalence of intracranial hemorrhage, and less need for red blood cell (RBC) transfusions (eTable 4 in the Supplement).

### ROP Prediction Using ROPScore

Using the training data set, the mean AUC, accuracy, sensitivity, and specificity were 0.76 (95% CI, 0.72-0.80), 69.0% (95% CI, 65.6%-72.3%), 61.7% (95% CI, 54.3%-68.7%), and 71.4% (95% CI, 67.4%-75.1%), respectively, for ROPScore to predict ROP occurrence and 0.67 (95% CI, 0.59-0.75), 67.8% (95% CI, 64.4%-71.2%), 53.7% (95% CI, 41.2%-65.8%), and 75.9% (95% CI, 66.9%-83.1%), respectively, for ROPScore to predict severe ROP (Table 2). Using the external validation data set, the mean AUC, accuracy, sensitivity, and specificity were 0.76 (95% CI, 0.72-0.80), 58.9% (95% CI, 55.3%-62.5%), 100% (95% CI, 83.4%-100%), and 43.1% (95% CI, 31.1%-55.9%), respectively, for ROPScore to predict ROP occurrence and 0.80 (95% CI, 0.62-0.98), 60.0% (95% CI, 56.4%-63.6%), 100% (95% CI, 59.8%-100%), and 41.2% (95% CI, 19.4%-66.5%), respectively, for ROPScore to predict severe ROP (Table 2).

### Visualization Saliency Map

The saliency map helps highlight areas used by the DL system for ROP prediction. The heat in saliency maps represents the importance of the regions on retinal photographs for the final prediction. A redder color indicates a greater contribution to the DL system; a bluer color indicates a smaller contribution to the DL system. For OC-Net, the saliency map mainly highlighted the optic disc and the vessels and areas around the optic disc. For SE-Net, the saliency map mainly highlighted the nasal and inferior areas around the optic disc (eFigure 4 in the Supplement). These results suggested that the fundus features of the optic disc and surrounding areas might have certain predictive value in ROP development.

### Weight Ratios of Different Characteristics

To identify the most important clinical characteristics in the DL system, we calculated the weight ratios of all characteristics in the last fully connected layer of OC-Net and SE-Net. The results identified GA, BW, use of RBC transfusion, use of oxygen in mechanical ventilation, and occurrence of respiratory distress syndrome (total weight ratios ≥36%) as the 5 most critical characteristics for OC-Net to predict ROP occurrence. BW, GA, cesarean delivery, use of RBC transfusion, and occurrence of intraventricular hemorrhage (total weight ratios ≥34%) were the 5 most critical characteristics for SE-Net to predict severe ROP (eFigure 2 in the Supplement).

## Discussion

In the present study, a DL system was developed to predict the occurrence and severity of ROP before 45 weeks’ PMA based on retinal photographs from the first screening and clinical characteristics collected before or at the first screening. Our results indicated that ROP prediction using the DL system with the major voting scheme was promising. With the help of our DL system, ophthalmologists and parents could be alerted to ensure that regular screening is performed before the onset of ROP. This DL system may also be applied to reduce the workload for pediatric ophthalmologists and to increase the treatment rate of severe ROP.

Some researchers have used regression analysis to predict ROP based on prenatal and postnatal factors.^16,20,21,22,23^ These prediction models that can identify infants with high risk of developing sight-threatening ROP early may improve the efficacy of ROP screening. However, retinal status is also important for ROP prediction. Previous studies have shown that minor vascular dilation, vascular tortuosity insufficiency in plus disease, the extent of temporal vascularization, and retinal immaturity at the first screening have prognostic significance in the early course of ROP.^25,26,27^ Thus, our DL system was developed to predict occurrence and severity of ROP using retinal photographs from the first screening along with clinical characteristics. Our results show that the integration of retinal and other clinical information using DL technology can achieve reliable accuracy in ROP prediction. In addition, we compared the birth characteristics between the false-negative and true-positive results for predicting the occurrence and severity of ROP (eTable 3 and eTable 4 in the Supplement). This investigation will help us better understand the characteristics of infants whose ROP diagnosis is missed or whose severity is incorrectly predicted and develop strategies to minimize the risk of misdiagnosis in the future.

To select the best output pattern of the DL system, we adopted 3 different training schemes. The majority voting scheme may be more similar to the clinical reality than the others, given that ophthalmologists make a diagnosis of ROP according to all retinal photographs taken in screenings. However, the 1-vote veto method may be used when high sensitivity is required, such as infants with multiple major risk factors for ROP. We also validated and compared the predictive performance of ROPScore with the DL system under the requirement of 100% sensitivity. The specificity and accuracy of SE-Net and ROPScore were comparable in predicting severe ROP.

Our DL system has 2 promising outcomes. First, early identification of infants with high risk of ROP, especially severe ROP, may prompt ophthalmologists and parents to perform fundus screening, which can reduce the risk of infants with severe ROP being misdiagnosed and untreated because of interrupted follow-up, thereby further reducing childhood blindness and societal burden.^13,14,15^ That was also why we classified ROP cases into mild and severe categories, so more alerts would be raised if the prediction outcome was severe ROP. Second, the global cost of ROP screening remains high, especially in remote areas.^12,14,18^ Regional nurseries without pediatric ophthalmologists typically transfer high-risk infants to tertiary hospitals for screening, which will incur substantial costs and delay treatment delivery.^11,12,35^ However, screening programs using digital photography and telemedicine can improve the cost-effectiveness of the health system and reduce the risk of infant transport.^11,12,35^

To minimize the black-box effect and increase model transparency,^30,31^ we used Grad-CAM to generate saliency maps to highlight the potential regions in retinal photographs from the first screening that contributed most toward ROP prediction. Notably, the saliency maps showed that OC-Net predicted ROP occurrence mainly according to the optic disc and the vessels and areas around the optic disc, while SE-Net predicted severe ROP mainly according to the nasal and inferior areas around the optic disc (eFigure 4 in the Supplement). These exploratory findings might help advance our understanding of the pathophysiological mechanisms and evolution of vascular abnormalities in the course of ROP. Previous studies have demonstrated decreased global average, nasal, and superior disc retinal nerve fiber layer (RNFL) thicknesses in preterm children and thinner average RNFL thickness in children with severe ROP.^56,57^ Therefore, the fundus features of the optic disc and surrounding areas may have a certain predictive value in ROP prediction. Additionally, the DL algorithms could identify and interpret pathological features that physicians could not perceive, regardless of their clinical experience.

In the future, our DL system may be integrated into cloud computing platforms for telemedicine ROP screening. After uploading retinal photographs from the first screening and clinical characteristics of an infant through a website, an automated ROP prediction could be generated. After an initial prediction report is completed, human experts would evaluate it online and generate a final report with a personalized follow-up scheme and a clinical decision.

### Limitations

Our study has some limitations. First, the generalizability of the DL system may be limited by the differences in the prevalence of clinical characteristics between different studies. Our DL system needs to be further validated by other prospective multiple-center data sets. Second, the DL system can only predict the occurrence of ROP and whether it is mild or severe ROP. More advanced DL systems that can provide more detailed predictions (eg, plus disease, stage, and zone) need to be developed in the future.

## Conclusions

In this study, our DL system provided accurate predictions of occurrence and severity of ROP before 45 weeks’ PMA based on retinal photographs from the first ROP screening and clinical characteristics measured before or at the first screening. The DL system is potentially helpful to identify infants at high risk of developing ROP and reduce blindness resulting from ROP.

## Notes

Supplement. **eAppendix 1.** Retinopathy of Prematurity Screening and Follow-up Schedule **eAppendix 2. **References, Detailed Definitions, and Time Point of Collection of the Clinical Characteristics **eAppendix 3. **Deep Learning System Development and Validation **eAppendix 4. **Prediction of Retinopathy of Prematurity Using ROPScore **eAppendix 5. **Improvement in Deep Learning System Performance by Clinical Characteristics **eFigure 1. **Distributions of Birth Weight and Gestational Age of All Included Infants **eFigure 2. **Weight Ratios of Different Characteristics **eFigure 3. **Calibration Plots for Evaluating the Calibration Ability of the Deep Learning System Using Major Voting Scheme **eFigure 4. **Original Retinal Photographs and Saliency Maps of Normal and Retinopathy of Prematurity Cases from Grad-CAM **eTable 1. **Comparison of Birth Characteristics Between the Included and Excluded Infants **eTable 2. **Performance of the Deep Learning System Using Different Training Schemes for Predicting Occurrence and Severity of Retinopathy of Prematurity in Internal Validation **eTable 3. **Comparison of Birth Characteristics of Infants With False-Negative and True-Positive Results for Prediction of Occurrence of Retinopathy of Prematurity **eTable 4. **Comparison of Birth Characteristics of Infants With False-Negative and True-Positive Results for Prediction of Severity of Retinopathy of Prematurity
eReferences.
