# Supplementary material for: Development and Validation of a Deep Learning Model to Predict the Occurrence and Severity of Retinopathy of Prematurity
Source: JAMA Netw Open. 2022 Jun 16;5(6):e2217447. doi: 10.1001/jamanetworkopen.2022.17447 (PMC10881218; doi:10.1001/jamanetworkopen.2022.17447)
Supplement: Supplement. — eAppendix 1. Retinopathy of Prematurity Screening and Follow-up Schedule eAppendix 2. References, Detailed Definitions, and Time Point of Collection of the Clinical Characteristics eAppendix 3. Deep Learning System Development and Validation eAppendix 4. Prediction of Retinopathy of Prematurity Using ROPScore eAppendix 5. Improvement in Deep Learning System Performance by Clinical Characteristics eFigure 1. Distributions of Birth Weight and Gestational Age of All Included Infants eFigure 2. Weight Ratios of Different Characteristics eFigure 3. Calibration Plots for Evaluating the Calibration Ability of the Deep Learning System Using Major Voting Scheme eFigure 4. Original Retinal Photographs and Saliency Maps of Normal and Retinopathy of Prematurity Cases from Grad-CAM eTable 1. Comparison of Birth Characteristics Between the Included and Excluded Infants eTable 2. Performance of the Deep Learning System Using Different Training Schemes for Predicting Occurrence and Severity of Retinopathy of Prematurity in Internal Validation eTable 3. Comparison of Birth Characteristics of Infants With False-Negative and True-Positive Results for Prediction of Occurrence of Retinopathy of Prematurity eTable 4. Comparison of Birth Characteristics of Infants With False-Negative and True-Positive Results for Prediction of Severity of Retinopathy of Prematurity eReferences. [file jamanetwopen-e2217447-s001.pdf]

Supplemental Online Content

Wu Q, Hu Y, Mo Z, et al. Development and validation of a deep learning model to predict the occurrence and severity of retinopathy of prematurity. *JAMA Netw Open.* 2022;5(6):e2217447. doi:10.1001/jamanetworkopen.2022.17447

eAppendix 1. Retinopathy of Prematurity Screening and Follow-up Schedule

eAppendix 2. References, Detailed Definitions, and Time Point of Collection of the Clinical Characteristics

eAppendix 3. Deep Learning System Development and Validation

eAppendix 4. Prediction of Retinopathy of Prematurity Using ROPScore

eAppendix 5. Improvement in Deep Learning System Performance by Clinical Characteristics

eFigure 1. Distributions of Birth Weight and Gestational Age of All Included Infants

eFigure 2. Weight Ratios of Different Characteristics

eFigure 3. Calibration Plots for Evaluating the Calibration Ability of the Deep Learning System Using Major Voting Scheme

eFigure 4. Original Retinal Photographs and Saliency Maps of Normal and Retinopathy of Prematurity Cases from Grad-CAM

eTable 1. Comparison of Birth Characteristics Between the Included and Excluded Infants

eTable 2. Performance of the Deep Learning System Using Different Training Schemes for Predicting Occurrence and Severity of Retinopathy of Prematurity in Internal Validation

eTable 3. Comparison of Birth Characteristics of Infants With False-Negative and True-Positive Results for Prediction of Occurrence of Retinopathy of Prematurity

eTable 4. Comparison of Birth Characteristics of Infants With False-Negative and True-Positive Results for Prediction of Severity of Retinopathy of Prematurity

eReferences.

This supplemental material has been provided by the authors to give readers additional information about their work.

© 2022 Wu Q et al. *JAMA Network Open.*

eAppendix 1. Retinopathy of Prematurity Screening and Follow-up Schedule

All included infants completed continuous follow-up until 45 weeks of postmenstrual age (PMA) and underwent serial ophthalmoscopic examinations under pupillary dilation according to the current screening guidelines.[1] According to the current screening guidelines, infants with gestational age (GA) <28 weeks received the initial retinal examinations at 31 weeks of PMA, and infants with GA ≥28 weeks had their first retinal examination at four weeks postnatal age.[1] Follow-up was scheduled according to the current screening guidelines: infants with type II ROP or suspected aggressive posterior-retinopathy of prematurity (ROP) need ≤one-week follow-up, infants with Zone II stage 2 ROP without plus disease need one- to two-week follow-up, infants with Zone II stage 1 ROP need two-week follow-up, and infants with Zone III any stage ROP need two- to three-week follow-up.[1]

© 2022 Wu Q et al. *JAMA Network Open.*

eAppendix 2. References, Detailed Definitions, and Time Point of Collection of the Clinical Characteristics

References Related to the Clinical Characteristics

We selected these clinical characteristics according to the reported risk factors associated with development of ROP identified by previous studies. All 46 clinical characteristics of each infant can be extracted from electronic medical records conveniently, including:

1) 7 maternal factors: maternal age,[2] intrauterine infection, cesarean delivery,[3,4] in vitro fertilization and embryo transfer treatment,[5] use of dexamethasone,[6] and occurrence of gestational hypertension,[7] and gestational diabetes.[8]

2) 18 neonatal factors: gender,[9] BW, GA,[10,11] infant small for GA,[12,13] 1-min and 5-min Apgar scores,[14] multiple or single gestation,[15] and occurrence of asphyxia, bronchopulmonary dysplasia,[16] pneumonia, intrauterine fetal distress, respiratory distress syndrome,[17] intraventricular hemorrhage,[18,19] sepsis,[20] hypoxic-ischemic encephalopathy,[19] necrotizing enterocolitis,[21] neonatal jaundice,[22] and patent ductus arteriosus.[23]

3) 7 treatment factors: history of oxygen exposure,[24] use and duration of oxygen in mechanical ventilation,[25,26] need for blood/RBC transfusion, volume and number of RBC transfusion.[27,28]

4) 14 laboratory factors: high-sensitivity C-reactive protein, WBC count,[29] RBC count, hemoglobin concentration,[27,28] hematocrit, corpuscular volume, RBC distribution width, absolute neutrophil count,[30] absolute lymphocyte count,[31] absolute monocyte count, platelet count, platelet volume, platelet distribution width,[32,33] and serum total bilirubin.[22]

Definitions of the Clinical Characteristics

Gestational age is defined according to the American Academy of Pediatrics' issued policy.[34] Small for GA is defined as the age-adjusted birth weight was below the 10th percentile according to a national survey in China.[35] Gestational hypertension is defined as new hypertension (systolic blood pressure ≥140 mmHg, or diastolic blood pressure ≥90 mmHg on 2 occasions, at least 4 hours apart) presenting after 20 weeks' gestation without clinically relevant proteinuria.[36] Gestational diabetes is defined as any degree of glucose intolerance with onset or first recognition during pregnancy.[37] Intraventricular hemorrhage was defined as ≥ grade 3 according to the Papile criteria.[38] Sepsis was defined as positive blood or cerebrospinal fluid culture and antibiotic therapy or intent of antibiotic therapy for 5 days or longer.[39] Bronchopulmonary dysplasia was defined as ventilation or oxygen dependency at 36 weeks' corrected age or at discharge, transfer, or death before 36 weeks.[40] Necrotizing enterocolitis was defined according to Bell criteria.[41] Other gestational diseases and neonatal disorders were diagnosed by certified obstetricians and neonatologists in the Zhujiang Hospital of Southern Medical University and Second Nanning People's Hospital.

Clinical Characteristics Collection Time Points

All maternal factors and several neonatal factors (i.e., gender, BW, GA, infant small for GA, 1-min and 5-min Apgar scores, and multiple or single gestation) were diagnosed and collected at the time point of infants' birth. The remaining neonatal factors (i.e., occurrence of asphyxia, bronchopulmonary dysplasia, intraventricular hemorrhage, sepsis, hypoxic-ischemic encephalopathy, respiratory distress syndrome, pneumonia, necrotizing enterocolitis, neonatal jaundice, patent ductus arteriosus, and intrauterine fetal distress) and all treatment factors were diagnosed and recorded between birth and the first ROP screening. All laboratory factors (mean white blood cell count, RBC count, hemoglobin concentration, hematocrit, corpuscular volume, RBC distribution width, absolute neutrophil count, absolute lymphocyte count, absolute monocyte count, platelet count, platelet volume, platelet distribution width, and serum total bilirubin) were routinely collected within the first 12h of life.[42] All blood chemistry tests were performed based on the first blood sample collected from each infant.

© 2022 Wu Q et al. *JAMA Network Open.*

eAppendix 3. Deep Learning System Development and Validation

Images Preprocessing

In the steps of multiple images preprocessing, saturated pixels with an intensity value of 255 or more in the retinal photographs were discarded, and the block-matching and 3D filtering method was employed to denoise and smooth the retinal photographs. All retinal photographs of each case were then resized into 256*256 pixel for residual network (ResNet)-50 (Microsoft Research).[43]

Deep Feature Extraction and Feature Vector Construction

In the ResNet-50, convolutional parameter layers were used for iteratively filters learning to transform input images into hierarchical feature maps, and to learn discriminative features at varying spatial levels without the need for manually tuned parameters. These convolutional layers were continuously positioned, whereby each layer transformed the input image to propagate the output information into the next layer. Finally, the learned deep features were extracted from the global average pooling layer that represented the average activations of each unit in this layer, yielding 512 features. The 512 highly abstracted features of each retinal photograph were concatenated with 46 clinical characteristics of the same case into a final vector of 558 dimensions for each retinal photograph.

Prediction Model Training

A deep neural network (DNN) was trained on our representative feature vector of 558 values to generate the predictive probability of each retinal photograph. We adopted 3 different training schemes to output the predictive labels based on the probability threshold of 0.30 of occurrence-network (OC-Net) and 0.45 of severity-network (SE-Net), respectively. The prediction label was 1 for the annotation of “ROP” and 0 for the annotation of “normal” in the OC-Net. The prediction label was 1 for the annotation of “severe ROP” and 0 for the annotation of “mild ROP” in the SE-Net.

First, under the majority voting method, we calculated the mean predictive probability of all retinal photographs from the same case. The prediction label of each case was divided into 1 (mean predictive probability equal to or larger than probability threshold) or 0 (mean predictive probability smaller than probability threshold). Second, under the one-vote veto method, we divided the prediction label of each retinal photograph to be 1 (predictive probability equal to or larger than probability threshold) or 0 (predictive probability smaller than probability threshold). If the prediction label of any retinal photograph in a case was 1, this case would be labeled as 1. Third, under the image-level method, we used the ground truth label of each case as the ground truth label of all corresponding retinal photographs. Meanwhile, we defined the prediction label of each retinal photograph to be 1 (predictive probability equal to or larger than probability threshold) or 0 (predictive probability smaller than probability threshold), and evaluated the prediction accuracy on an image level. Through the above the 3 training schemes, patient-level and image-level predictive labels could be respectively obtained at this stage. The Pytroch codes used in this manuscript can be found on GitHub at https://github.com/yaoMYZ/ROP.

Cross-validation on the Training Set Using Different Training Schemes

The 5-fold cross-validation manner was used for internal validation of the 3 training schemes. In this manner, the occurrence-dataset and the severity-dataset were both randomly and equally divided into 5 independent sub-samples, 4 of which were used to train the OC-Net and SE-Net of each training scheme, respectively, with the remaining one used as for internal validation and fine tuning. This procedure was repeated until each sub-sample had been used as the validation set. Among the 3 training schemes, both OC-Net and SE-Net of the major voting scheme achieved the best overall performance (eTable 2 in the Supplement). Thus, the major voting scheme was deployed in the deep learning system for further validation under requirement of 100% sensitivity.

© 2022 Wu Q et al. *JAMA Network Open.*

eAppendix 4. Prediction of Retinopathy of Prematurity Using ROPScore

ROPScore is accessible online and also used to predict occurrence and severe type of retinopathy of prematurity (ROP) for each infant before 45 weeks of postmenstrual age.[44,45] Linear coefficients (β) of birth weight (BW), gestational age (GA), proportional weight gain at completed 6 weeks of life, use of mechanical ventilation, and need for blood transfusions was -0.004, -0.263, -1.258, +1.920, and +1.980, respectively.[44,45] These β were applied as weighted values in the final equation: 24.847-0.004*BW-0.263*GA-1.258* proportional weight gain+1.920* use of mechanical ventilation (insert 1 if the baby used oxygen-therapy under mechanical ventilation before the 6th week of life or insert 0 if not)+1.980* need for blood transfusions (insert 1 if the baby received blood transfusion before the 6th week of life or insert 0 if not).[44,45] The suggested alarm cut-off score is 11 and 14.5 for occurrence and severe type of ROP, respectively.[44,45]

© 2022 Wu Q et al. *JAMA Network Open.*

eAppendix 5. Improvement in Deep Learning System Performance by Clinical Characteristics

The performance of the deep learning system under 100% sensitivity requirement was evaluated when only using retinal photographs from the first retinopathy of prematurity (ROP) screening to predict ROP in the internal validation. With the major voting scheme, mean areas under the receiver operating characteristic curve (AUC), accuracy, sensitivity, and specificity was 0.60 (95% CI: 0.57-0.64), 26.1% (95% CI: 22.9%-29.3%), 100% (95% CI: 97.4%-100%), and 1.5% (95% CI: 0.7%-3.0%), respectively, in predicting ROP occurrence, and was 0.46 (95% CI, 0.38-0.53), 41.4% (95% CI, 34.3%-48.5%), 100% (95% CI, 93.2%-100%), and 2.1% (95% CI, 0.7%-5.1%), respectively, in predicting severe ROP. Comparing the results with those of using both retinal photographs and clinical characteristics, we found that clinical characteristics to predict ROP occurrence could improve the AUC by 50.0%, the accuracy by 102.3%, and the specificity by 2420%. Moreover, clinical characteristics to predict severe ROP could improve the AUC by 89.1%, the accuracy by 64.3%, and the specificity by 2119%.

© 2022 Wu Q et al. *JAMA Network Open.*

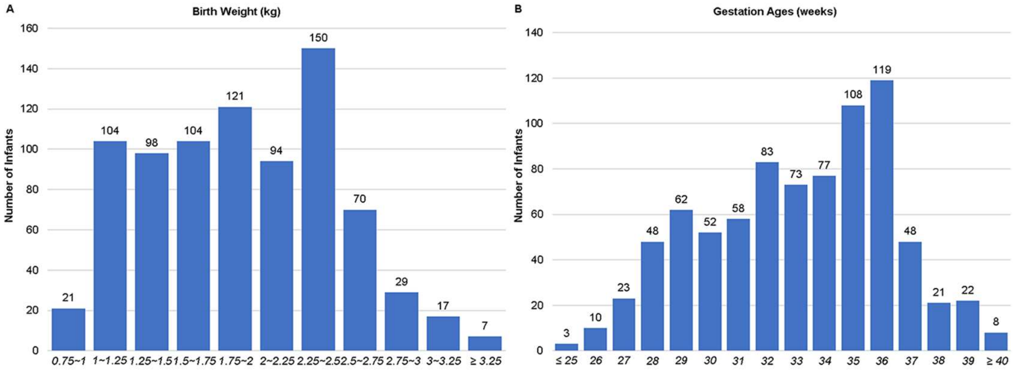

eFigure 1. Distributions of Birth Weight and Gestational Age of All Included Infants

A, Each bar represents the number of infants whose birth weight were within the given range. B, Each bar represents the number of infants whose gestation age were within the given weeks.

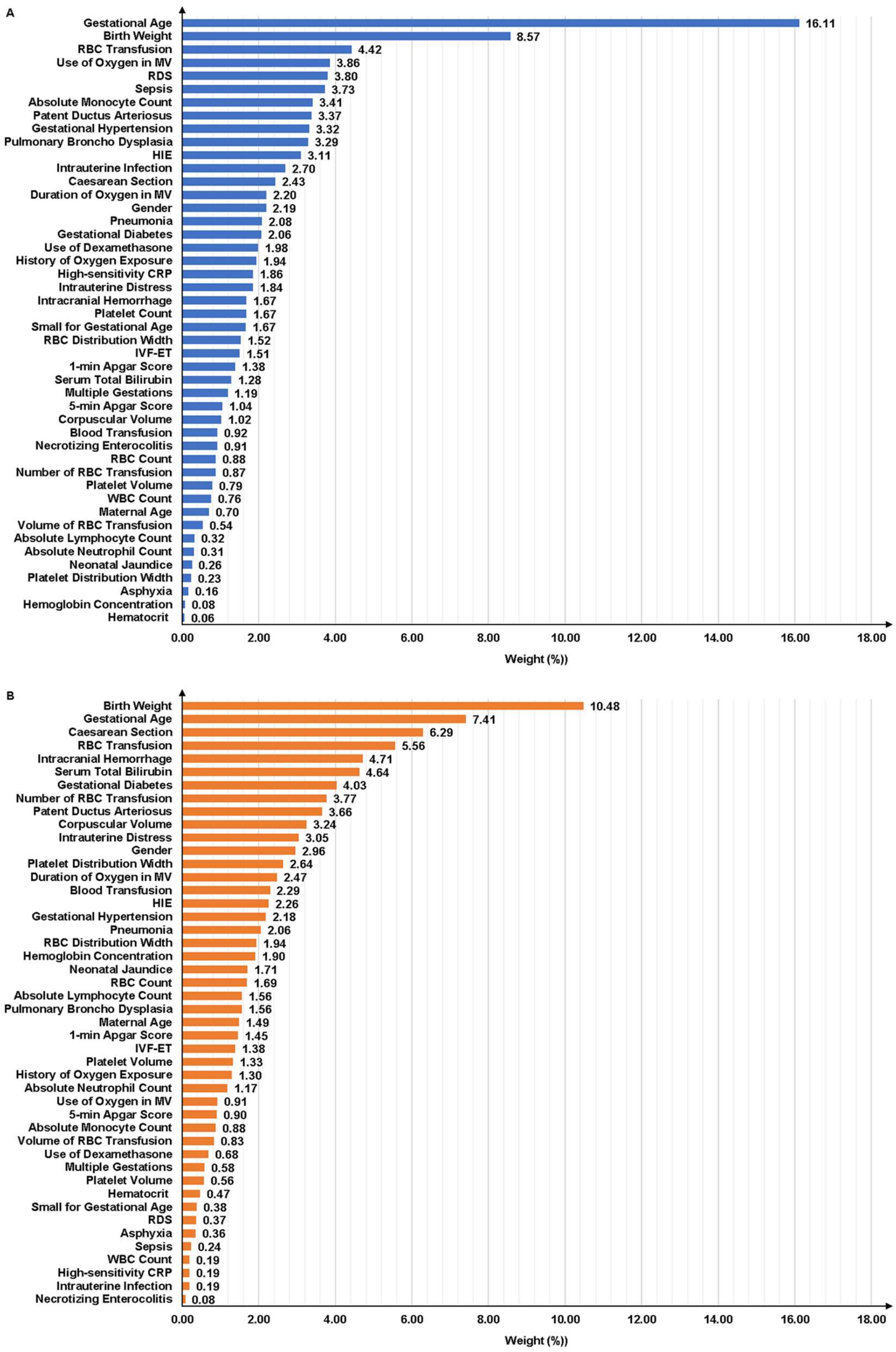

eFigure 2. Weight Ratios of Different Characteristics

The absolute values of the weights of each characteristic in the last fully-connected layers of the deep learning (DL) system were summed to obtain the corresponding importance factors. Then, the importance factors of all 46 characteristic were normalized to obtain the final weight ratios. The bar of the final weight ratios indicates the importance of different features as average for the DL system on 5 test runs. A, The blue bars represent the weight ratios of the OC-Net. B, The orange bars represent the weight ratios of the SE-Net. The higher the bar is, the more important the corresponding clinical characteristics is for the prediction task. Abbreviations: RBC, red blood cell; MV, mechanical ventilation; RDS, respiratory distress syndrome; HIE, hypoxic ischemic encephalopathy; CRP, C-reactive protein; IVF-ET, in vitro fertilization and embryo transfer; WBC, white blood cell.

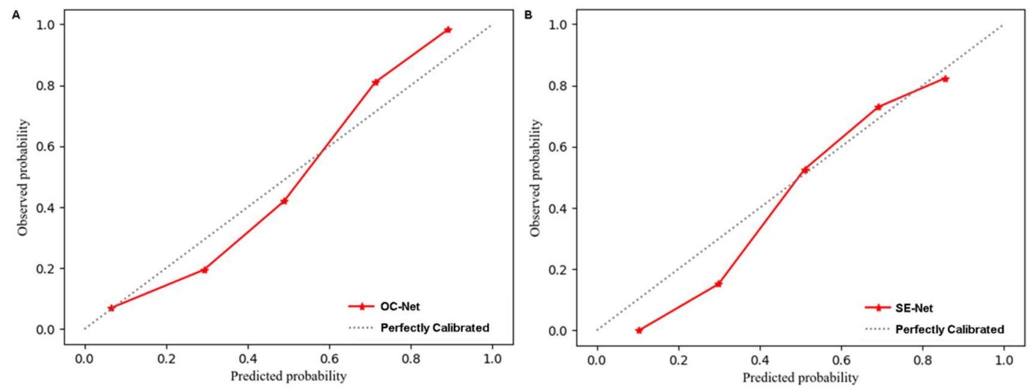

eFigure 3. Calibration Plots for Evaluating the Calibration Ability of the Deep Learning System Using Major Voting Scheme

Calibration plots for the observed proportion of occurrence and severe type of retinopathy of prematurity versus predictive probability are obtained from OC-Net (A) and SE-Net (B), respectively.

© 2022 Wu Q et al. *JAMA Network Open.*

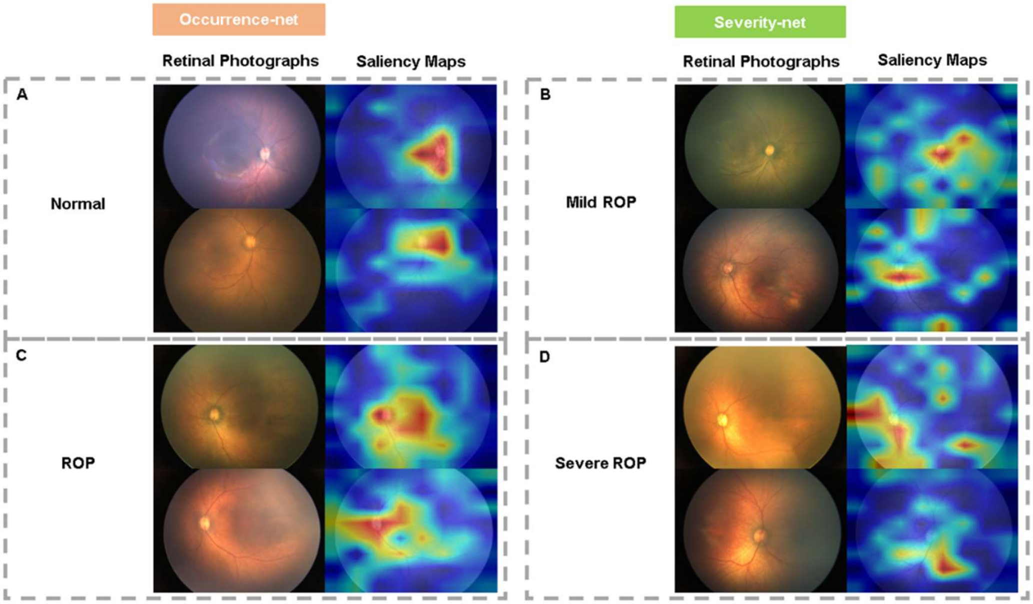

eFigure 4. Original Retinal Photographs and Saliency Maps of Normal and Retinopathy of Prematurity Cases from Grad-CAM

Grad-CAM is used to generate color saliency heat-maps that highlight the regions playing important roles in the final prediction of occurrence-network (A and B) and severity-network (C and D) under the major voting scheme. In the saliency maps, the red regions indicate a stronger contribution than the green regions, and the blue regions have low to no contribution to the retinopathy of prematurity prediction.

© 2022 Wu Q et al. *JAMA Network Open.*

eTable 1. Comparison of Birth Characteristics Between the Included and Excluded Infants

**Characteristics**	**Included Infants (n=815)**	**Excluded Infants (n=173)**	***P* value**
Boys, n (%)	450 (55.2)	87 (50.3)	0.237[a]
Gestational Age, mean (SD), weeks	33.1 (3.2)	32.0 (4.1)	0.102[b]
≤28 weeks	84 (10.3%)	26 (15.0%)	0.073[a]
29-32 weeks	255 (31.3%)	46 (26.6%)	0.223[a]
≥33 weeks	476 (58.4%)	101 (58.4%)	0.995[a]
Birth Weight, mean (SD), kg	1.91 (0.6)	1.87 (0.6)	0.373[b]
0.75-1.5 kg	223 (27.4%)	52 (30.1%)	0.472[a]
1.5-2.0 kg	225 (27.6%)	43 (24.9%)	0.460[a]
2.0-2.5 kg	244 (29.9%)	56 (32.4%)	0.528[a]
≥2.5 kg	123 (15.1%)	22 (12.7%)	0.423[a]
Small for Gestational Age, No. (%)	61 (7.5)	16 (9.2)	0.432[a]
Multiple Gestations, No. (%)	202 (24.8)	35 (20.2)	0.203[a]

[a] Chi-square Test, [b] Unpaired Mann-Whitney Test. Abbreviations: SD standard deviation.

© 2022 Wu Q et al. *JAMA Network Open.*

eTable 2. Performance of the Deep Learning System Using Different Training Schemes for Predicting Occurrence and Severity of Retinopathy of Prematurity in Internal Validation

	AUC	Accuracy (%)	Sensitivity (%)	Specificity (%)
Prediction of Retinopathy of Prematurity Occurrence				
OC-Net (Major Voting, 95% CI)	0.90 (0.88-0.92)	76.9 (73.8-80.0)	88.6 (82.8-92.6)	73.1 (69.1-76.7)
OC-Net (One-vote Veto, 95% CI)	0.81 (0.78-0.84)	77.9 (74.9-80.9)	86.0 (79.7-90.3)	75.3 (71.4-78.8)
OC-Net (Image-level, 95% CI)	0.91 (0.90-0.92)	77.2 (74.1-80.3)	86.5 (80.3-90.8)	73.6 (69.7-77.2)
Prediction of Severe Retinopathy of Prematurity				
SE-Net (Major Voting, 95% CI)	0.87 (0.82-0.91)	78.5 (72.5-84.5)	88.0 (77.3-94.3)	73.0 (64.1-80.9)
SE-Net (One-vote Veto, 95% CI)	0.80 (0.74-0.86)	78.0 (72.0-84.0)	90.9 (79.1-95.3)	69.2 (59.6-77.0)
SE-Net (Image-level, 95% CI)	0.83 (0.82-0.84)	76.3 (70.1-82.5)	86.2 (73.8-92.2)	69.9 (60.5-77.8)

Abbreviations: AUC, area under the receiver operating characteristic curve; OC-Net, occurrence-network; SE-Net, severity-network; CI confidence interval.

© 2022 Wu Q et al. *JAMA Network Open.*

eTable 3. Comparison of Birth Characteristics of Infants With False-Negative and True-Positive Results for Prediction of Occurrence of Retinopathy of Prematurity

Characteristics	False Negative Infants (n=21)	True Positive Infants (n=187)	*P* value
**Maternal Factors**			
Maternal Age, mean (SD), years	31.5 (3.9)	29.8 (5.5)	0.145[b]
Caesarean Section, No. (%)	15 (71.4)	71 (38.0)	0.003[a]
IVF-ET, No. (%)	2 (9.5)	22 (11.8)	1.000[a]
Gestational Hypertension, No. (%)	0 (0.0)	6 (3.2)	1.000[a]
Gestational Diabetes, No. (%)	4 (19.0)	29 (15.5)	0.752[a]
Intrauterine Infection, No. (%)	2 (9.5)	2 (1.1)	0.052[a]
Use of Dexamethasone, No. (%)	3 (14.3)	42 (22.5)	0.577[a]
**Neonatal Factors**			
Gestational Age, mean (SD), weeks	34.2 (2.1)	29.1 (2.0)	$<$ 0.001[b]
Birth Weight, mean (SD), kg	2.12 (0.42)	1.34 (0.35)	$<$ 0.001[b]
Boys, No. (%)	10 (47.6)	103 (55.1)	0.515[a]
Small for Gestational Age, No. (%)	2 (9.5)	11 (5.9)	0.626[a]
Multiple Gestations, No. (%)	6 (28.6)	42 (22.5)	0.529[a]
1-min Apgar Score, mean (SD)	8.6 (2.5)	7.7 (2.2)	0.003[b]
5-min Apgar Score, mean (SD)	9.5 (1.4)	8.8 (1.6)	0.003[b]
Intrauterine Distress, No. (%)	2 (9.5)	19 (10.2)	1.000[a]
Asphyxia, No. (%)	3 (14.3)	57 (30.5)	0.136[a]
Pulmonary Broncho Dysplasia, No. (%)	1 (4.8)	94 (50.3)	$<$ 0.001[a]
Intracranial Hemorrhage, No. (%)	3 (14.3)	77 (41.2)	0.018[a]
Sepsis, No. (%)	1 (4.8)	34 (18.2)	0.213[a]
Hypoxic Ischemic Encephalopathy, No. (%)	1 (4.8)	48 (25.7)	0.031[a]
Respiratory Distress Syndrome, No. (%)	5 (23.8)	110 (58.8)	0.002[a]
Pneumonia, No. (%)	7 (33.3)	128 (68.4)	0.001[a]
Necrotizing Enterocolitis, No. (%)	2 (9.5)	19 (10.2)	1.000[a]
Neonatal Jaundice, No. (%)	16 (76.2)	124 (66.3)	0.360[a]
Patent Ductus Arteriosus, No. (%)	6 (28.6)	71 (38.0)	0.398[a]
**Treatment Factors**			
History of Oxygen Exposure, No. (%)	14 (66.7)	181 (96.8)	$<$ 0.001[a]
Use of Oxygen in MV, No. (%)	9 (42.9)	165 (88.2)	$<$ 0.001[a]

© 2022 Wu Q et al. *JAMA Network Open.*

eTable 3. Comparison of Birth Characteristics of Infants With False-Negative and True-Positive Results for Prediction of Occurrence of Retinopathy of Prematurity (continued)

Characteristics	False-negative Infants (n=21)	True-positive Infants (n=187)	*P* value
Duration of Oxygen in MV, mean (SD), days	12.0 (22.3)	31.7 (24.3)	$< 0.001^b$
Blood Transfusion, n (%)	10 (47.6)	157 (84.0)	$< 0.001^a$
RBC Transfusion, n (%)	7 (33.3)	149 (79.7)	$< 0.001^a$
Number of RBC Transfusion, mean (SD), n	0.9 (1.9)	2.3 (2.1)	$< 0.001^b$
Volume of RBC Transfusion, mean (SD), U	0.31 (0.66)	0.79 (0.76)	$< 0.001^b$
**Laboratory Factors**			
High-sensitivity CRP, mean (SD), mg/L	3.7 (10.8)	6.3 (17.0)	0.304^b
WBC Count, mean (SD), 10^9/L	11.4 (5.1)	11.4 (6.6)	0.735^b
RBC Count, mean (SD), 10^{12}/L	4.4 (0.9)	4.1 (1.0)	0.039^b
Hemoglobin Concentration, mean (SD), g/L	153.1 (28.6)	135.5 (33.4)	0.012^b
Hematocrit, mean (SD), mg/L	0.45 (0.09)	0.40 (0.10)	0.007^b
Corpuscular Volume, mean (SD), fL	104.1 (5.3)	98.4 (11.9)	0.045^b
RBC Distribution Width, mean (SD), %	15.9 (1.1)	16.8 (2.3)	0.119^b
Absolute Neutrophil Count, mean (SD), 10^9/L	9.4 (14.4)	6.3 (5.2)	0.341^b
Absolute Lymphocyte Count, mean (SD), 10^9/L	2.9 (1.0)	3.8 (4.2)	0.251^b
Absolute Monocyte Count, mean (SD), 10^9/L	1.2 (0.5)	1.6 (1.9)	0.316^b
Platelet Count, mean (SD), 10^9/L	227.9 (88.6)	263.2 (125.0)	0.300^b
Platelet Volume, mean (SD), fL	9.7 (2.3)	10.5 (1.2)	0.060^b
Platelet Distribution Width, mean (SD), %	12.5 (3.2)	12.5 (2.5)	0.524^b
Serum Total Bilirubin, mean (SD), μmol/L	97.6 (53.3)	110.8 (77.1)	0.659^b

[a] Chi-square Test, [b] Unpaired Mann-Whitney Test. Abbreviations: SD standard deviation.

Abbreviations: IVF-ET, in vitro fertilization and embryo transfer; MV, mechanical ventilation; CRP, C-reactive protein; RBC, red blood cell; WBC, white blood cell; SD standard deviation.

© 2022 Wu Q et al. *JAMA Network Open.*

eTable 4. Comparison of Birth Characteristics of Infants With False-Negative and True-Positive Results for Prediction of Severity of Retinopathy of Prematurity

Characteristics	False Negative Infants (n=10)	True Positive Infants (n=65)	*P* value
**Maternal Factors**			
Maternal Age, mean (SD), years	28.7 (6.5)	29.8 (5.6)	0.497[b]
Caesarean Section, No. (%)	6 (60.0)	16 (24.6)	0.055[a]
IVF-ET, No. (%)	3 (30.0)	7 (10.8)	0.124[a]
Gestational Hypertension, No. (%)	0 (0.0)	1 (1.5)	1.000[a]
Gestational Diabetes, No. (%)	1 (10.0)	11 (16.9)	1.000[a]
Intrauterine Infection, No. (%)	0 (0.0)	1 (1.5)	1.000[a]
Use of Dexamethasone, No. (%)	3 (30.0)	12 (18.5)	0.408[a]
**Neonatal Factors**			
Gestational Age, mean (SD), weeks	27.5 (1.1)	27.7 (1.3)	0.772[b]
Birth Weight, mean (SD), kg	1.53 (0.56)	1.13 (0.22)	0.026[b]
Boys, No. (%)	6 (60.0)	36 (55.4)	1.000[a]
Small for Gestational Age, No. (%)	1 (10.0)	9 (13.8)	1.000[a]
Multiple Gestations, No. (%)	3 (30.0)	12 (18.5)	0.408[a]
1-min Apgar Score, mean (SD)	6.2 (3.5)	7.5 (2.2)	0.361[b]
5-min Apgar Score, mean (SD)	8.0 (2.1)	8.5 (1.9)	0.488[b]
Intrauterine Distress, No. (%)	2 (20.0)	6 (9.2)	0.288[a]
Asphyxia, No. (%)	5 (50.0)	20 (30.8)	0.230[a]
Pulmonary Broncho Dysplasia, No. (%)	5 (50.0)	38 (58.5)	0.615[a]
Intracranial Hemorrhage, No. (%)	2 (20.0)	38 (58.5)	0.038[a]
Sepsis, No. (%)	2 (20.0)	16 (24.6)	1.000[a]
Hypoxic Ischemic Encephalopathy, No. (%)	3 (30.0)	32 (49.2)	0.321[a]
Respiratory Distress Syndrome, No. (%)	7 (70.0)	44 (67.7)	1.000[a]
Pneumonia, No. (%)	7 (70.0)	48 (73.8)	1.000[a]
Necrotizing Enterocolitis, No. (%)	2 (20.0)	6 (9.2)	0.288[a]
Neonatal Jaundice, No. (%)	6 (60.0)	50 (76.9)	0.262[a]
Patent Ductus Arteriosus, No. (%)	5 (50.0)	18 (27.7)	0.154[a]
**Treatment Factors**			
History of Oxygen Exposure, No. (%)	9 (90.0)	64 (98.5)	0.250[a]
Use of Oxygen in MV, No. (%)	7 (70.0)	60 (92.3)	0.068[a]

© 2022 Wu Q et al. *JAMA Network Open.*

eTable 4. Comparison of Birth Characteristics of Infants With False-Negative and True-Positive Results for Prediction of Severity of Retinopathy of Prematurity (continued)

Characteristics	False Negative Infants (n=10)	True Positive Infants (n=65)	*P* value
Duration of Oxygen in MV, mean (SD), days	35.7 (33.5)	32.7 (22.6)	0.895[b]
Blood Transfusion, n (%)	8 (80.0)	61 (93.8)	0.180[a]
RBC Transfusion, n (%)	8 (80.0)	60 (90.7)	0.233[a]
Number of RBC Transfusion, mean (SD), n	1.9 (1.7)	3.0 (2.3)	0.181[b]
Volume of RBC Transfusion, mean (SD), U	0.62 (0.52)	0.93 (0.73)	0.325[b]
**Laboratory Factors**			
High-sensitivity CRP, mean (SD), mg/L	2.0 (2.7)	8.8 (21.2)	0.925[b]
WBC Count, mean (SD), 10^9/L	12.6 (3.2)	10.3 (5.1)	0.019[b]
RBC Count, mean (SD), 10^{12}/L	3.8 (1.0)	3.9 (0.79)	0.493[b]
Hemoglobin Concentration, mean (SD), g/L	125.9 (42.7)	128.5 (29.0)	0.478[b]
Hematocrit, mean (SD), mg/L	0.38 (0.12)	0.38 (0.09)	0.743[b]
Corpuscular Volume, mean (SD), fL	100.4 (9.2)	96.2 (11.0)	0.207[b]
RBC Distribution Width, mean (SD), %	17.0 (1.9)	16.8 (2.1)	0.685[b]
Absolute Neutrophil Count, mean (SD), 10^9/L	6.6 (3.0)	5.5 (4.0)	0.108[b]
Absolute Lymphocyte Count, mean (SD), 10^9/L	4.4 (1.2)	3.9 (1.3)	0.110[b]
Absolute Monocyte Count, mean (SD), 10^9/L	1.5 (0.9)	1.3 (0.7)	0.362[b]
Platelet Count, mean (SD), 10^9/L	243.4 (96.9)	261.6 (104.7)	0.418[b]
Platelet Volume, mean (SD), fL	10.7 (1.3)	10.4 (1.6)	1.000[b]
Platelet Distribution Width, mean (SD), %	12.9 (3.8)	12.6 (2.5)	0.797[b]
Serum Total Bilirubin, mean (SD), μmol/L	121.1 (68.2)	118.8 (85.2)	0.738[b]

[a] Chi-square Test, [b] Unpaired Mann-Whitney Test. Abbreviations: SD standard deviation.

Abbreviations: IVF-ET, in vitro fertilization and embryo transfer; MV, mechanical ventilation; CRP, C-reactive protein; RBC, red blood cell; WBC, white blood cell; SD standard deviation.

eReferences.

1. Fierson WM, Ophthalmology AAOPSo, American Academy Of O, American Association For Pediatric O, Strabismus, American Association Of Certified O. Screening Examination of Premature Infants for Retinopathy of Prematurity. *Pediatrics.* 2018;142(6).
2. Wu WC, Ong FS, Kuo JZ, et al. Retinopathy of prematurity and maternal age. *Retina.* 2010;30(2):327-331.
3. Manzoni P, Farina D, Maestri A, et al. Mode of delivery and threshold retinopathy of prematurity in pre-term ELBW neonates. *Acta Paediatr.* 2007;96(2):221-226.
4. Darlow BA, Hutchinson JL, Henderson-Smart DJ, et al. Prenatal risk factors for severe retinopathy of prematurity among very preterm infants of the Australian and New Zealand Neonatal Network. *Pediatrics.* 2005;115(4):990-996.
5. Minasian M, Fielder A. IVF babies with ROP at higher gestational age and birth weight: implications of changing screening criteria. *Br J Ophthalmol.* 2005;89(8):1066.
6. Travers CP, Clark RH, Spitzer AR, Das A, Garite TJ, Carlo WA. Exposure to any antenatal corticosteroids and outcomes in preterm infants by gestational age: prospective cohort study. *BMJ.* 2017;356:j1039.
7. Zayed MA, Uppal A, Hartnett ME. New-onset maternal gestational hypertension and risk of retinopathy of prematurity. *Invest Ophthalmol Vis Sci.* 2010;51(10):4983-4988.
8. Tunay ZO, Ozdemir O, Acar DE, Oztuna D, Uras N. Maternal Diabetes as an Independent Risk Factor for Retinopathy of Prematurity in Infants With Birth Weight of 1500 g or More. *Am J Ophthalmol.* 2016;168:201-206.
9. Pivodic A, Hard AL, Lofqvist C, et al. Individual Risk Prediction for Sight-Threatening Retinopathy of Prematurity Using Birth Characteristics. *JAMA Ophthalmol.* 2019;138(1):1-9.
10. Hellstrom A, Smith LE, Dammann O. Retinopathy of prematurity. *Lancet.* 2013;382(9902):1445-1457.
11. Hansen RM, Moskowitz A, Akula JD, Fulton AB. The neural retina in retinopathy of prematurity. *Prog Retin Eye Res.* 2017;56:32-57.
12. Dhaliwal CA, Fleck BW, Wright E, Graham C, McIntosh N. Retinopathy of prematurity in small-for-gestational age infants compared with those of appropriate size for gestational age. *Arch Dis Child Fetal Neonatal Ed.* 2009;94(3):F193-195.
13. Allegaert K, Vanhole C, Casteels I, et al. Perinatal growth characteristics and associated risk of developing threshold retinopathy of prematurity. *J AAPOS.* 2003;7(1):34-37.
14. Sjobom U, Hellstrom W, Lofqvist C, et al. Analysis of Brain Injury Biomarker Neurofilament Light and Neurodevelopmental Outcomes and Retinopathy of Prematurity Among Preterm Infants. *JAMA Netw Open.* 2021;4(4):e214138.
15. Barr S, Poggi S, Keszler M. Triplet morbidity and mortality in a large case series. *J Perinatol.* 2003;23(5):368-371.
16. Podraza W, Michalczuk B, Jezierska K, et al. Correlation of Retinopathy of Prematurity with Bronchopulmonary Dysplasia. *Open Med (Wars).* 2018;13:67-73.
17. Lad EM, Nguyen TC, Morton JM, Moshfeghi DM. Retinopathy of prematurity in the United States. *Br J Ophthalmol.* 2008;92(3):320-325.

© 2022 Wu Q et al. *JAMA Network Open.*

18. Watts P, Adams GG, Thomas RM, Bunce C. Intraventricular haemorrhage and stage 3 retinopathy of prematurity. *Br J Ophthalmol.* 2000;84(6):596-599.
19. Kella YR, Snir M, Ehrlich R, et al. Time to normalization of intracranial pressure secondary to intraventricular hemorrhage and the need for retinopathy of prematurity treatment in infants diagnosed with both conditions. *J AAPOS.* 2012;16(6):515-517.
20. Manzoni P, Maestri A, Leonessa M, Mostert M, Farina D, Gomirato G. Fungal and bacterial sepsis and threshold ROP in preterm very low birth weight neonates. *J Perinatol.* 2006;26(1):23-30.
21. Coskun Y, Dalkan C, Yabas O, et al. A predictive score for retinopathy of prematurity by using clinical risk factors and serum insulin-like growth factor-1 levels. *Int J Ophthalmol.* 2017;10(11):1722-1727.
22. Weintraub Z, Carmi N, Elouti H, Rumelt S. The association between stage 3 or higher retinopathy of prematurity and other disorders of prematurity. *Can J Ophthalmol.* 2011;46(5):419-424.
23. Tsui I, Ebani E, Rosenberg JB, Lin J, Angert RM, Mian U. Patent ductus arteriosus and indomethacin treatment as independent risk factors for plus disease in retinopathy of prematurity. *J Pediatr Ophthalmol Strabismus.* 2013;50(2):88-92.
24. Holmstrom G, Tornqvist K, Al-Hawasi A, Nilsson A, Wallin A, Hellstrom A. Increased frequency of retinopathy of prematurity over the last decade and significant regional differences. *Acta Ophthalmol.* 2018;96(2):142-148.
25. Kim TI, Sohn J, Pi SY, Yoon YH. Postnatal risk factors of retinopathy of prematurity. *Paediatr Perinat Epidemiol.* 2004;18(2):130-134.
26. Allegaert K, de Coen K, Devlieger H, EpiBel Study G. Threshold retinopathy at threshold of viability: the EpiBel study. *Br J Ophthalmol.* 2004;88(2):239-242.
27. Inder T, Clemett R, Austin N, Graham P, Darlow BJTJop. High iron status in very low birth weight infants is associated with an increased risk of retinopathy of prematurity. *J Pediatr.* 1997;131(4):541-544.
28. Dani C, Reali M, Bertini G, Martelli E, Pezzati M, Rubaltelli FJEhd. The role of blood transfusions and iron intake on retinopathy of prematurity. *Early Hum Dev.* 2001;62(1):57-63.
29. Rivera JC, Dabouz R, Noueihed B, Omri S, Tahiri H, Chemtob S. Ischemic Retinopathies: Oxidative Stress and Inflammation. *Oxid Med Cell Longev.* 2017;2017:3940241.
30. Kurtul BE, Kabatas EU, Zenciroglu A, et al. Serum neutrophil-to-lymphocyte ratio in retinopathy of prematurity. *J AAPOS.* 2015;19(4):327-331.
31. Christensen RD, Baer VL, Gordon PV, et al. Reference ranges for lymphocyte counts of neonates: associations between abnormal counts and outcomes. *Pediatrics.* 2012;129(5):e1165-1172.
32. Lundgren P, Lundberg L, Hellgren G, et al. Aggressive Posterior Retinopathy of Prematurity Is Associated with Multiple Infectious Episodes and Thrombocytopenia. *Neonatology.* 2017;111(1):79-85.
33. Jensen AK, Ying GS, Huang J, Karp K, Quinn GE, Binenbaum G. Thrombocytopenia and retinopathy of prematurity. *J AAPOS.* 2011;15(1):e3-e4.

© 2022 Wu Q et al. *JAMA Network Open.*

34. Engle WA, American Academy of Pediatrics Committee on F, Newborn. Age terminology during the perinatal period. *Pediatrics.* 2004;114(5):1362-1364.
35. Li N, Li Z, Ye R, et al. Preconception Blood Pressure and Risk of Low Birth Weight and Small for Gestational Age: A Large Cohort Study in China. *Hypertension.* 2016;68(4):873-879.
36. Visintin C, Mugglestone MA, Almerie MQ, et al. Management of hypertensive disorders during pregnancy: summary of NICE guidance. *BMJ.* 2010;341:c2207.
37. American Diabetes A. Diagnosis and classification of diabetes mellitus. *Diabetes Care.* 2014;37 Suppl 1:S81-90.
38. Papile L, Burstein J, Burstein R, Koffler HJTJop. Incidence and evolution of subependymal and intraventricular hemorrhage: a study of infants with birth weights less than 1,500 gm. *J Pediatr.* 1978;92(4):529-534.
39. Stoll BJ, Hansen N, Fanaroff AA, et al. Late-onset sepsis in very low birth weight neonates: the experience of the NICHD Neonatal Research Network. *Pediatrics.* 2002;110(2 Pt 1):285-291.
40. Jobe AH, Bancalari E. Bronchopulmonary dysplasia. *Am J Respir Crit Care Med.* 2001;163(7):1723-1729.
41. Bell MJ, Ternberg JL, Feigin RD, et al. Neonatal necrotizing enterocolitis. Therapeutic decisions based upon clinical staging. *Ann Surg.* 1978;187(1):1-7.
42. Fortes Filho JB, Dill JC, Ishizaki A, Aguiar WW, Silveira RC, Procianoy RS. Score for Neonatal Acute Physiology and Perinatal Extension II as a predictor of retinopathy of prematurity: study in 304 very-low-birth-weight preterm infants. *Ophthalmologica.* 2009;223(3):177-182.
43. He K, Zhang X, Ren S, Sun J. Deep Residual Learning for Image Recognition. Paper presented at: 2016 IEEE Conference on Computer Vision and Pattern Recognition (CVPR); 27-30 June 2016, 2016.
44. Piermarocchi S, Bini S, Martini F, et al. Predictive algorithms for early detection of retinopathy of prematurity. *Acta Ophthalmol.* 2017;95(2):158-164.
45. Eckert GU, Fortes Filho JB, Maia M, Procianoy RS. A predictive score for retinopathy of prematurity in very low birth weight preterm infants. *Eye (Lond).* 2012;26(3):400-406.

© 2022 Wu Q et al. *JAMA Network Open.*
